# The Role of Artificial Intelligence in Imaging-Based Diagnosis of Retinal Dystrophy and Evaluation of Gene Therapy Efficacy

**DOI:** 10.3390/jpm15120605

**Published:** 2025-12-05

**Authors:** Weronika Chuchmacz, Barbara Bobowska, Alicja Forma, Eliasz Dzierżyński, Damian Puźniak, Barbara Teresińska, Jacek Baj, Joanna Dolar-Szczasny

**Affiliations:** 1 Department of Forensic Medicine, Medical University of Lublin, ul. Jaczewskiego 8b, 20-090 Lublin, Poland; 2 Department of Correct, Clinical and Imaging Anatomy, Medical University of Lublin, ul. Jaczewskiego 4, 20-090 Lublin, Poland; 60812@umlub.edu.pl; 3 Department of Plastic Surgery, St. John’s Cancer Center, ul. Jaczewskiego 7, 20-090 Lublin, Poland; 4 Department of General and Pediatric Ophthalmology, Medical University of Lublin, 20-093 Lublin, Poland

**Keywords:** retinal dystrophy, artificial intelligence, neural networks, gene therapy, computer-aided diagnosis (CAD), convolutional neural networks (CNN)

## Abstract

**Introduction:** Inherited retinal dystrophies (IRDs) are genetically determined conditions leading to progressive vision loss. Developments in gene therapy are creating new treatment options for IRD, but require precise imaging diagnosis and monitoring. According to recent studies, artificial intelligence, especially deep neural networks, could become an important tool for analyzing imaging data. **Material and Methods:** A systematic literature review was conducted in accordance with PRISMA guidelines, using PubMed, Scopus, and Web of Science databases to identify publications from 2015 to 2025 on the application of artificial intelligence in diagnosing inherited retinal dystrophies and monitoring the effects of gene therapy. The included articles passed a two-stage selection process and met the methodological quality criteria. **Results:** Among all the included studies it can be noticed that the use of artificial intelligence in diagnostics and therapy of IRDs is rather effective. The most common method was deep learning with its subtype convolutional neural networks (CNNs). However, there is still a place for improvement due to various limitations occurring in the studies. **Conclusions:** The review points to the growing potential of AI models in optimizing the diagnostic and therapeutic pathway in IRDs, while noting current limitations such as low data availability, the need for clinical validation, and the interpretability of the models. AI may play a key role in personalized ophthalmic medicine in the near future, supporting both clinical decisions and interventional study design.

## 1. Background

Retinal dystrophies are a group of eye diseases that are characterized by progressive damage to the retina, which over time can lead to vision loss. Hereditary retinal dystrophies (IRDs) are rooted in genetic abnormalities, caused by mutations in one of more than 280 different genes responsible for visual function [1,2]. Inheritance of the disease can be autosomal dominant, recessive, X-chromosome coupled, and mitochondrial [3,4]. The condition is the most common cause of hereditary blindness, affecting an average of 1 in 1500–2000 people in the general population [1].

Retinal dystrophies can involve both the central (macula) and peripheral parts, with the entire retinal surface being affected in advanced stages of the disease. Classification of these conditions can be based on the type of photoreceptors affected (rods, cones, or both at the same time), the clinical picture, or the degree of retinal atrophy [2]. IRDs include retinal pigmentary degeneration, cone-rod dystrophy, juvenile macular degeneration (AMD), choroideremia (CHIM), and Usher syndrome [3].

IRDs are neurodegenerative conditions with a wide spectrum of symptoms and varying rates of disease progression and severity in different patients [4]. Among the most common symptoms are decreased visual acuity, impaired night vision, metamorphopsia, impaired color perception, and loss of peripheral visual field [5].

Patients with hereditary retinal diseases show significant and progressive deterioration of visual function, which can be monitored by a number of diagnostic tools, such as visual acuity measurement, visual field assessment, electroretinography (ERG), structural imaging using autofluorescence, spectral-domain optical tomography (SD-OCT), and OCT angiography (OCTA) [4,5].

Although modern, non-invasive imaging methods enable an accurate clinical diagnosis, genetic testing plays a key role in confirming a specific phenotype. In addition, genetic segregation analysis makes it possible to accurately determine the pattern of inheritance. Many cases of IRD are monogenic or digenic in origin, but the same gene can account for a variety of clinical pictures, indicating the complexity of the relationship between genotype and phenotype [4,5,6].

**Figure 1:**
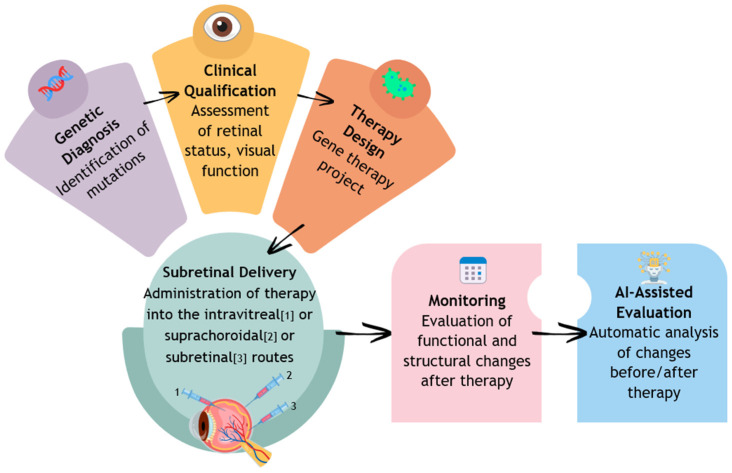
Diagnosis, implementation of treatment, and monitoring of the course of retinal dystrophy.

Currently, a promising form of IRD treatment is gene integration therapy. This method involves treating a genetic defect by delivering a correct copy of the damaged gene to retinal cells. Most commonly, viral vectors are used, which have significant affinity for retinal cells while being low in immunogenicity. Once the vector is introduced into the target cells, the inserted gene is transcribed, leading to the synthesis of a functional protein, which ultimately leads to the inhibition of disease progression or partial improvement in photoreceptor function. The main targets of this therapy are the outer retinal photoreceptors and retinal pigment epithelial (RPE) cells (Figure 1) [7,8].

Artificial intelligence (AI) is playing an increasingly important role in ophthalmology. The application of AI in the diagnosis of IRD covers a wide range of activities, including utility in diagnostic testing to make a diagnosis, monitoring the course of the disease, and predicting response to treatment. AI opens up new possibilities for automatic analysis of retinal images and interpretation of genetic data. This is particularly useful in the context of IRDs, which are rare diseases characterized by genetic heterogeneity, which can significantly improve the availability of specialized evaluation and accelerate the implementation of targeted genetic diagnosis [9].

Methods for diagnosing and monitoring retinal dystrophy include visual acuity assessment, perimetry, electroretinography, or structural imaging techniques (OCT, Fundus Autofluorescence—FAF, OCTA). The tests provide valuable clinical data, but the interpretation of this information can be time-consuming, subjective, and difficult to detect for minimal changes and deviations. In this context, AI, especially models based on deep learning (DL) and convolutional networks (CNNs), makes it possible to analyze retinal images automatically and with high accuracy, detect early lesions, and classify patients by disease type and severity. AI can also support the analysis of genetic data, which is particularly important in the case of IRD, where a single gene can determine different phenotypes, and many diseases are monogenic or digenic in origin. In addition, by integrating various data sources, imaging, clinical, and molecular, it becomes possible to create predictive models of disease progression to predict the rate of progression and potential response to treatment, including gene therapies [9,10].

The use of AI in IRD allows patients to be pre-qualified and shortens the time from symptom onset to diagnosis. In the long term, AI can also play a key role in personalizing treatment, allowing the frequency of follow-up visits, type of therapy, and monitoring strategies to be tailored to the individual patient profile. The integrated approach, combining clinical, genetic, and informatics expertise, lays the groundwork for a new generation of precision medicine in ophthalmology. The diagnostic capabilities of AI and IRD are presented in Table 1 (Table 1) [9,10,11].

In clinical practice, interpreting complex retinal imaging data remains challenging due to the high phenotypic variability and overlapping manifestations among different forms of IRD. This clinical heterogeneity complicates both accurate diagnosis and the establishment of genotype–phenotype correlations. In this context, AI-based methods offer valuable tools by enabling automated pattern recognition, quantitative image analysis, and consistent interpretation across large datasets. The potential of AI in genotype prediction, phenotype classification, and early detection of subtle retinal structural changes may significantly enhance diagnostic accuracy, reduce diagnostic delays, and improve patient selection for gene therapy.

The purpose of this review is to systematically identify, analyze, and synthesize the current state of knowledge on the application of artificial intelligence algorithms, particularly neural networks, in the imaging diagnosis of retinal dystrophy and in evaluating the effectiveness of gene therapy.

## 2. Materials and Methods

This review was conducted to provide a structured and comprehensive synthesis of an overview of the available knowledge on the application of AI in the imaging diagnosis of retinal dystrophy and monitoring the effectiveness of gene therapy. The systematic review was conducted in accordance with PRISMA 2020 (Preferred Reporting Items for Systematic Reviews and Meta-Analyses) guidelines (refer to Supplementary Table S1). The project has been registered in the PROSPERO database under the CRD420251234013.

Major databases were used for the literature review: PubMed (National Library of Medicine), Web of Science (Clarivate Analytics), and Scopus (Elsevier), to provide a comprehensive collection of peer-reviewed articles relevant to the topic. Publications from 2015–2025 were included to cite the latest developments in AI and the diagnosis, treatment, and monitoring of retinal dystrophy. The review covers work from 2015–2025, as this period saw the emergence of studies combining imaging data with genetic analysis, as well as the first attempts to use AI to support gene therapy eligibility and monitor the effects of these therapies. This scope allows us to capture modern, clinically relevant methods and their evolution.

The search strategy included the use of specific keywords and complex queries combining terms related to artificial intelligence (“artificial intelligence,” “deep learning,” “neural networks”, “convolutional neural network”, “machine learning”, “generative adversarial networks”) with terms related to retinal dystrophy (“retinal dystrophy,” “inherited retinal dystrophies”, “retinal pigmentary degeneration”, “juvenile macular degeneration”) and imaging-based diagnosis and gene therapy (“diagnosis”, “imaging” “gene therapy”, “genotype”, “treatment monitoring”, “fundus”, “optical coherence tomography”). Logical operators (and, or, not) were used to narrow the search results.

Inclusion criteria were defined. The review included original prospective and retrospective studies evaluating the use of artificial intelligence, machine learning, or deep learning in the analysis of retinal images in the context of IR) and the qualification or monitoring of gene therapies. Studies involving humans with suspected or confirmed IRD, including clinical or cohort populations, were included. Studies reporting results on diagnostic accuracy (sensitivity, specificity, AUC), genotype prediction ability, phenotype classification, or detection of progression after gene therapy were included. The time frame of the publications covered the years 2015–2025, and the articles analyzed had to be published in English in medical journals.

Studies conducted exclusively on animal models or in vitro were excluded from the analysis as they do not reflect clinical conditions. Studies using only synthetic data or images generated without reference to patient data were also excluded. The gray literature without peer review, including conference abstracts without full data, technical reports, and preprints, was removed from the review. Case reports and small series (n ≤ 5) were also excluded from the main analysis. In the case of duplicates or analyses based on the same cohorts, only the most complete and up-to-date study was included to avoid double counting of patient data.

The article selection process consisted of two stages. Initially, titles and abstracts were evaluated to ensure that they met the inclusion criteria. Then the full texts of the selected papers were subjected to detailed analysis. The selection was carried out independently by two reviewers, and discrepancies were resolved by consulting a third expert. From the selected publications, data were extracted on: the type of retinal dystrophy, the AI algorithm used, imaging methods, diagnostic parameters, as well as gene therapy monitoring methods and study results. The quality of the included studies was assessed using QUADAS-2.

**Figure 2:**
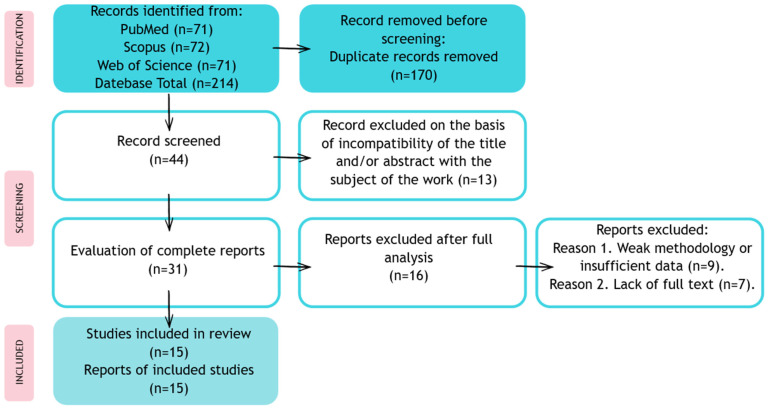
Publication review process and inclusion in a systematic review.

The results of the study were presented in the form of a narrative synthesis, with a particular focus on the differences in the algorithms and imaging methods used. The analysis made it possible to identify current trends, barriers, and prospects for the development of the use of AI in the diagnosis and therapy of retinal dystrophy. The review and inclusion of studies are summarized in Figure 2.

## 3. Results

### 3.1. Convolutional Neural Networks (CNNs)

Deep learning is a subfield of machine learning inspired by the way the human brain works. Classic ML algorithms require manual feature engineering. Deep learning automatically learns feature representations at multiple levels of abstraction: earlier layers learn low-level features, and subsequent layers learn more complex and semantic ones. This allows it to achieve excellent results in image, sound, and text processing, among other things [12].

Convolutional neural networks are the most popular and effective deep learning architecture, widely used in image analysis. Their main advantage is the ability to automatically detect and recognize important features in visual data without the need to manually define them. CNN’s operation is based on convolutional layers, which process the image through filters that capture various patterns, such as edges or textures. Subsequent layers learn increasingly complex representations, which enables the network to accurately recognize objects or structures. Due to this architecture, CNNs are extremely effective in tasks such as image classification, segmentation, and object detection, and are widely used in fields such as medicine, facial recognition, and autonomous vehicles [13].

In Fujinami-Yokokawa et al.’s 2019 study, it was emphasized that the application of deep neural networks in the prediction of the three major genes responsible for IRDs (*ABCA4*, *RP1L1*, *EYS*) from SD-OCT had a mean prediction accuracy of 90% [14]. The test effectiveness for *ABCA4* was at ~100% and *RP1L1* and *EYS* tests had a sensitivity and specificity of ≥85–100%. This suggests that deep learning on OCT images can support genetic diagnostics in ophthalmology, accelerating disease identification and enabling earlier interventions, but because of the small dataset further study is needed [14].

According to the Fujinami-Yokokawa et al.’s 2021 multicenter study, the method of deep learning used on retinal images from fundus photographs and FAF can be an effective tool to help detect and identify genetic eye diseases [15]. Achieving over 80% accuracy suggests that this technology can enter wider clinical practice, speeding up diagnostics and facilitating treatment. Also, with the use of deep learning techniques, the cost and number of unnecessary genetic tests can be reduced. However, authors emphasize the limitations of using deep neural networks in IRDs which, among others, are a small cohort size and a large number of genetic mutations that can cause IRDs [15].

A high accuracy of using artificial intelligence was also found in Kominami et al.’s study, where convolutional neural networks were observed to classify the level of central vision loss in retinitis pigmentosa (RP) using FAF images and color fundus images [16]. The accuracy of the CNN models for color fundus images was lower than for FAF images. It was found that the CNN model was especially good at recognizing severe damage. In comparison with clinical evaluation, the model showed promising effectiveness, although it sometimes confused mild cases with normal images. The authors acknowledged the rarity of RP and the importance of further studies [16].

In another study about using deep learning methods to detect RP based on color fundus photographs, Chen et al. used three models of transfer learning (Inception V3, Inception Resnet V2, and Xception), with the best performance of the Xception [17]. It is highlighted that the AI model had the highest values of accuracy (96%), sensitivity, and precision compared to the results of ophthalmology experts, which suggests it can support early diagnosis, especially in areas with limited ophthalmological resources or in telemedicine [17].

Deep learning method, particularly CNN, was also tested to classify broader cases of IRDs, including Stargardt disease (STGD), Best disease (BD), and retinitis pigmentosa using FAF images in the Miere et al. study. It resulted in an overall accuracy of 95%, with high sensitivity and specificity for all disease groups. However, the limitations of this study include the small size of both the training and test sets, the test being limited to only three hereditary diseases, and the risk of over-learning (data leakage), because the images may come from the same eyes at different stages [18].

AI can potentially speed up diagnosis, improve accessibility, and lower costs, which will enable earlier and targeted genetic treatment. This was tested by Pontikos et al., who introduced the algorithm, Eye2Gene, which is composed of multiple deep convolutional neural networks and can differentiate up to 63 genes responsible for IRD based on images (SD-OCT, FAF, IR) [19]. The model is trained on a multimodal dataset of images with IRDs. The authors indicate that Eye2Gene owes its effectiveness to the fact that it was developed based on one of the world’s largest datasets. In an internal test (Moorfields Eye Hospital), the model achieved a “top-5” accuracy of 83.9%—meaning it found the correct gene among the 5 most suggested genes more often than experts. It has been externally validated in 5 centers (Oxford, Liverpool, Bonn, Tokyo, São Paulo) with effectiveness confirmed in various populations and equipment [19].

Analysis of the results in Table 2 confirms the growing effectiveness of artificial intelligence methods in the diagnosis of IRD (Table 2).

Convolutional models based on InceptionV3, ResNet, and Xception achieved high accuracy (81–96%) in the classification of IRD genotypes and phenotypes, with the highest results obtained in studies with large datasets and in autofluorescence analysis [16,17,18]. The introduction of multimodal approaches, such as Eye2Gene, has enabled the prediction of disease genes based on FAF, IR, and OCT images, which is a breakthrough towards personalized qualification for gene therapy [19]. Taken together, these data indicate that the integration of artificial intelligence with image analysis can significantly improve the diagnosis and monitoring of IRDs.

Comparing the performance of AI models across different imaging modalities, such as OCT, FAF, or fundus photography, is a crucial step in assessing their practical usefulness in diagnosing IRD. The differences in the characteristics of the acquired data translate into varying effectiveness of the algorithms, which allows for identifying the most effective approaches and potential limitations of each imaging method.

Table 3 compares the results of several studies that used machine learning algorithms to recognize retinal diseases based on different types of images (OCT, FAF, fundus) (Table 3).

The number of images, type of disease, sensitivity, specificity, and accuracy of the models were taken into account. The analyzed models show very high effectiveness in classifying retinal diseases in OCT, FAF, and fundus images. The highest and most stable results appear in studies with a larger amount of data, which highlights the importance of large training sets [20]. Very high-accurate results in small trials may suggest the risk of overfitting or limited generalization [14]. FAF seems to be a slightly more demanding modality than OCT, although it still provides very high effectiveness. Moreover, the comparison of FAF and fundus images showed similar results in both image types. In general, artificial intelligence shows great potential in the diagnosis of genetic retinal diseases, but larger, more diverse datasets and multi-center validation are needed to confirm the possibility of broad clinical use.

### 3.2. Generative Adversarial Networks (GANs)

Generative adversarial networks are a deep learning model in which two interacting neural networks—the so-called generator and discriminator—function in a rivalry system. The task of the generator is to create artificial data, such as retinal images, so that the generated data become negative training examples for the discriminator. The discriminator, on the other hand, is responsible for assessing the reliability of the data, distinguishing false data generated by the generator from real data [21].

**Figure 3:**
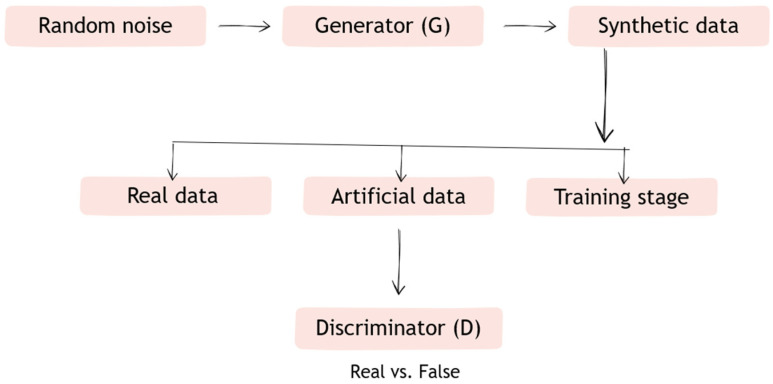
Diagram of how Generative adversarial networks (GANs) work.

GANs show great potential in ophthalmology, especially in generating synthetic data, improving image quality, and predicting treatment outcomes. Their application can support diagnosis and treatment planning and monitoring. However, a limitation remains the insufficient variety of training data, which affects the realism of the generated images. Advances in this field include the integration of physiological knowledge with GAN architecture, such as by mapping retinal blood flow. With the increasing availability of open-source data and tools, the technology is poised for wider clinical application (Figure 3) [22].

Description of operation:

- Step 1. The generator (G) receives a random vector and generates data (e.g., retinal images).
- Step 2. The discriminator (D) receives both the data generated by G and real data (e.g., from a medical database).
- Step 3. D learns to distinguish between synthetic and real data, and G learns to create data in such a way as to “fool” D.
- Step 4. The networks learn simultaneously—the generator becomes better at creating realistic data, and the discriminator becomes better at recognizing it.

The study by Veturi et al. used the StyleGAN2-ADA model to generate synthetic autofluorescence (FAF) images representing different IRD phenotypes. The aim of the study was to evaluate the applicability of synthetic data in improving IRD diagnosis using GAN by increasing the variety and amount of training data for AI models analyzing retinal imaging. The use of SynthEye enabled the generation of synthetic IRD images characterized by high visual fidelity, with analogous diversity to real images, excluding duplicates. The results of the study indicated that the synthetic images improved classification performance, with no performance degradation, and reduced the risk of overfitting due to better representation of rare IRD variants [23].

Kamran et al. conducted a study to develop a new, precise method for retinal macro- and microvascular segmentation in retinal images, given its key role in determining retinal degenerative diseases. They developed the Retinal Vessel GAN (RV-GAN) model, which uses two generators and two multiscale discriminators to increase the precision of vascular detection and segmentation. In addition, it introduces weighted feature matching loss to increase the importance of information from the discriminator decoder, which is responsible for assessing the realism of images at the pixel level, and integrates this loss into classical image reconstruction to lead to better preservation of vascular structure during model learning. The proposed architecture achieved very high AUCs of 0.9887 (DRIVE), 0.9914 (CHASE-DB1), and 0.9887 (STARE), confirming that RV-GAN effectively detects both large and small retinal vessels, outperforming previous approaches. This test is potentially important for the development of computer-aided diagnosis tools in ophthalmology. Accurate vascular segmentation can support earlier detection of retinal diseases, assessment of lesion progression, and the development of artificial intelligence-based systems to support the diagnosis of genetic eye diseases [24].

Schlegl et al. investigated the utility of the AnoGAN models and the f-AnoGAN version in the process of detecting anomalies in retinal OCT images, without the need for prior pathology determination. By applying the model to retinal OCT images, they were able to identify areas containing retinal fluid and hyperreflective foci, lesions associated with IRD progression, and automatically evaluate image sections for their similarity to healthy retina. The results show that the model enables early detection of IRD changes and tracking of disease progression [25,26].

A study by Kamran et al. investigated the use of conditional generative opponent network (conditional GAN) as a non-invasive alternative to classic fluorescein angiography (FA), which is the standard method for diagnosing retinal vascular disease. The model achieved a low FID (Fréchet Inception Distance) score = 30.3, indicating the high quality of the generated images and their high statistical similarity to real angiograms. Qualitative evaluation showed that the images generated by the network were indistinguishable from real FA. Compared to other state-of-the-art generative methods, the proposed architecture achieved better results both quantitatively and visually, without the need for intravenous fluorescein dye [27].

A review of studies summarized in Table 4 highlights the expanding role of Generative Adversarial Networks (GANs) in diagnosing and monitoring inherited retinal diseases (IRD) (Table 4).

Early applications of StyleGAN2-ADA focused on generating synthetic FAF images to augment limited datasets, improving model robustness and reducing overfitting. RV-GAN demonstrated the highest accuracy (AUC 0.988–0.991) for precise vascular segmentation, supporting microangiopathy monitoring. Both AnoGAN and f-AnoGAN effectively detected subtle or subclinical OCT abnormalities (AUC ~0.85–0.92), facilitating early identification of atypical IRD forms. Moreover, conditional GANs (cGAN) enabled the prediction of fluorescein angiography (FA) images from standard fundus photographs (AUC 0.91–0.95), offering a non-invasive alternative for vascular assessment. Collectively, GAN-based approaches enhance data diversity and analytical precision, paving the way toward more efficient, image-driven monitoring of gene therapy outcomes in IRD.

## 4. Discussion

### 4.1. AI in Diagnosis of Retinal Dystrophy

The dynamic development of technology and intensive research into artificial intelligence point to its significant potential in streamlining clinical processes, improving population health outcomes, and reducing disparities in access to medical care. AI in ophthalmology can support diagnosis, disease monitoring, and clinical decision-making, increasing the accuracy and speed of patient assessment. Despite promising results, however, the implementation of AI in clinical practice remains limited, which undermines the real usefulness of these solutions [28].

Jafarbeglou et al. conducted a study to evaluate the effectiveness of a deep learning model with a multi-object architecture in detecting retinal pigmentary degeneration and Stargardt disease and in differentiating them from healthy eyes. The analysis covered 391 cases (158 RP, 62 STGD, and 171 controls), using an image database containing CFP and IR images. MobileNetV2 models with a single input for each imaging modality and a multi-modality model integrating both types of data (diagnostic accuracy 94.4% vs. 96.3%) were used. These results confirm that DL mechanisms achieve high performance in diagnosing IRD [29].

A study conducted by Mouiee et al. evaluated the ability of a convolutional neural network to classify histological images at different stages of retinal degeneration. The best-performing model achieved high agreement with expert assessments (F1: 85–90%, Cohen’s kappa: 0.86), and its performance was comparable to that of observers in terms of image interpretation. A comparison with six observers showed that the variability between the model and the observers was similar to the variability between the observers themselves. Restricting the image context resulted in a maximum 6% decrease in model performance, while reducing the number of training images decreased the model’s effectiveness by about 10% compared to the full set, highlighting the importance of adequate data quantity and image quality for classification reliability [30].

Miere et al. conducted a study evaluating the possibility of automatically classifying retinal atrophy according to etiology based on fundus autofluorescence images using a deep CNN. The model was trained using 251 FAF images from patients with advanced dry age-related macular degeneration—geographic atrophy (GA), and genetically confirmed IRD in the late stages of atrophy, i.e., Stargardt disease and pseudo-Stargardt’s optic dystrophy (PSPD). In the first approach, the model was tested on 63 untrained FAF images, achieving a maximum accuracy of 92% and an AUC-ROC of 0.981, with an average accuracy of 87.3 ± 2.96%. In the second approach, using 10-fold cross-validation, the average accuracy was 79 ± 6%. These results indicate that deep learning algorithms can distinguish GA and late-onset IRDs masquerading as GA on FAF with good accuracy and high AUC-ROC values [31].

Wang et al. conducted a study in which a CNN was used to automatically identify the ellipsoidal zone (EZ) in OCT scans of patients with retinitis pigmentosa. A total of approximately 2.87 million image fragments obtained from 480 B-scans in both RP and healthy individuals were used for the study. The model achieved high accuracy in the classification of individual retinal layers, including 91% for the EZ, and showed a strong correlation with expert measurements (r = 0.97; *p* < 0.0001). Bland–Altman analysis showed that the differences between the EZ width measured by the model and the assessors’ measurements were comparable to the differences observed between the experts themselves [32].

The results of the presented research clearly indicate that generative adversarial networks are becoming an increasingly promising tool in modern ophthalmology, both in the area of diagnostics and in simulating the course of retinal diseases. The main advantage of these models is their ability to generate synthetic data with high visual fidelity, which can supplement limited collections of real clinical images. The problem of insufficient data, especially in the case of rare retinal dystrophies, remains a significant limitation for classical deep learning models [33].

Jeon et al. describe the k-SALSA method, created to generate synthetic retinal images that maintain diagnostic value while protecting patient privacy. Instead of using individual patient data, the system combines features of several images and generates an averaged version, ensuring k-anonymity. Technically, it is based on generative algorithms that combine local style and structure features from multiple images to create new, synthetic representations of the retina. The k-SALSA method achieves MIA (Membership Inference Attack) accuracy of 1% for the APTOS dataset and 0.52% for the EyePACS dataset, respectively, for k = 10. Tests showed that the generated images retain important clinical information and are resistant to identification attempts, making k-SALSA a promising tool for securely sharing data in medical research [34].

Wang et al. describe that using a StyleGAN2 model and a “human-in-the-loop” training approach, they were able to generate synthetic images of the fundus containing changes characteristic of AMD. The results showed that the evaluators were unable to reliably distinguish between the synthetic and real image (accuracy at 0.66), and that when using an objective realness scale, the accuracy of the evaluation increased to 0.72. This solution can support the development of diagnostic algorithms by increasing the diversity of training data and covering less common AMD changes. Although the authors note that clinical validation and improvement of synthetic image interpretability are still necessary [35].

Anaya-Sánchez et al. focus on using a gradient penalty Wasserstein Generative Adversarial Network with Gradient Penalty (WGAN-GP) to generate synthetic retinal images, which is particularly important due to the limited availability of medical data necessary to train artificial intelligence models in ophthalmology diagnostics. In the study, WGAN-GP was used to create realistic images of the retina that could serve as training data for other AI models. The results showed that the proposed method surpasses traditional generative models, such as conditional GANs and PathoGAN. Furthermore, expert evaluations showed that only 56.66% of synthetic images could be distinguished from real ones, which indicates the high fidelity and clinical usefulness of the generated data. The results confirm that this technique is a promising method for creating synthetic medical data, which may contribute to the development of more advanced diagnostic systems in ophthalmology [36].

### 4.2. AI in Evaluation of Gene Therapy Efficacy

Neurodegenerative diseases of the retina are characterized by limited treatment options, and current therapies mainly slow down their progression. The difficulties in developing effective therapeutic methods stem primarily from the high genetic diversity of IRDs, which prevents a thorough understanding of the mechanisms underlying this group of diseases. Therefore, gene-independent approaches based on network medicine, enabling better diagnosis, prognosis, and drug repositioning, are becoming increasingly important. CNNs show promising potential in identifying genes associated with IRDs, supporting genetic diagnosis, family counseling, and patient recruitment for clinical trials [37].

Pontikos et al. conducted the Eye2Gene study to identify genes responsible for retinal diseases based on OCT and infrared/FAF images. Fifteen CNN Inception V3 networks were used, trained on the largest IRD dataset to date (44,817 scans from 1907 patients) and verified on four independent datasets. The model achieved high performance in predicting the 63 most common IRD genes (AUC = 0.950) and outperformed clinical experts, with 82.2% accurate predictions compared to 75.3% for ophthalmologists [19,38].

The thickness of the outer nuclear layer (ONL) in OCT images is an important biomarker of photoreceptor preservation and gene therapy efficacy in patients with IRD. In response to the limitations of existing segmentation methods, Eckardt et al. developed an artificial intelligence algorithm based on the U-Net architecture, trained on images of healthy patients using domain adaptation for IRD data. The model achieved 98.7% accuracy in ONL segmentation and enabled the creation of retinal thickness maps to support quantitative assessment of gene therapy effects [39].

AI models offer the possibility of identifying the most likely gene mutations responsible for the occurrence of IRD, based on retinal imaging. This not only allows for earlier genetic diagnosis of patients, but also contributes to an increase in the percentage of people in whom the genetic cause can be detected, known as “diagnostic yield,” and enables the classification of patients for clinical trials. However, the models require good image quality and sufficient data for a given gene, which means that rarer genes are examined to a significantly lesser extent [19]. In addition, the use of AI models enables earlier detection of therapeutic effects. Thanks to the possibility of automatic and quantitative analysis of image data, it is possible to detect structural changes in the photoreceptor layers before a measurable improvement in visual function occurs. This allows for more sensitive and objective monitoring of treatment effectiveness in a shorter time after administration of the therapy. In addition, the use of artificial intelligence in generating repeatable, quantitative endpoints improves the quality and reliability of clinical trials by reducing measurement errors resulting from subjective assessment or inter-observer variability. This approach also makes it possible to reduce the size of research samples while maintaining high statistical power, which translates into more effective clinical trial design. In addition, automatic image analysis supports the process of selecting patients for gene therapy, i.e., individuals with better-preserved retinal structure, as assessed by deep learning segmentation models, may be classified as potentially better prognosis. As a result, AI-based tools support both the evaluation of gene therapy efficacy and the personalization of therapeutic decisions, increasing the precision and effectiveness of the entire treatment process [1,40].

## 5. Challenges and Limitations

Methods using artificial intelligence are a promising and innovative tool supporting the diagnosis and treatment of retinal diseases. However, their use is associated with certain limitations. The most serious challenge remains the rarity and heterogeneity of data. IRD diseases are rare by nature, and it is difficult to obtain large, well-described image collections and corresponding genotype data. Most of the available databases come from single centers, often with a limited ethnic population, which makes it difficult to create models with high generalization ability. As a result, models trained on such narrow datasets may not perform well in external populations [41].

Another limitation is the retrospective nature of most studies. Current AI analyses in IRD are mostly based on archival data, which makes it impossible to clearly demonstrate that decisions supported by algorithms translate into better clinical outcomes. Therefore, prospective clinical trials are needed to confirm in a controlled manner that the use of artificial intelligence actually improves the effectiveness of diagnosis, qualification, and monitoring of gene therapy. Regulatory and clinical acceptance issues also remain an important aspect. Any diagnostic or decision-making tool requires formal validation and certification before it can be used to make therapeutic decisions. This process is time-consuming and requires compliance with rigorous standards of quality, safety, and data transparency [42].

The use of AI algorithms in inherited retinal diseases remains limited, especially in the context of precisely differentiating between individual dystrophies and distinguishing them from other retinal pathologies, such as scars after laser surgery or previous choroiditis. The studies analyzed most often pointed to limitations such as small sample sizes, lack of ethnic diversity, elimination of lower-quality images, comparisons only with normal images, and the retrospective nature of the analyses. In addition, none of the studies to date have assessed the potential role of artificial intelligence in the field of genetic counseling. At this stage, the use of AI in IRD seems to be limited mainly to retinal structure segmentation and quantitative analysis [41].

Another serious issue is the so-called “black box” of many deep learning architectures, which generate diagnostic results without transparent justification or the ability to trace decision paths. This lack of interpretability remains a major obstacle to clinical trust and routine implementation. To address these challenges, future research should prioritize the development of understandable AI frameworks that enable visualization and understanding of the image features that influence model predictions [38].

## 6. Future Directions

Currently, artificial intelligence technology in the context of IRD is mainly in the preclinical research and validation phase, with predominant use in automatic retinal structure segmentation and quantitative analysis. However, there is a lack of large-scale, multicenter studies confirming the efficacy and safety of these solutions in everyday practice. This means that AI is not yet ready for routine clinical use in IRD diagnosis, but it is a rapidly growing area of translational research that may significantly impact the diagnostic process and patient eligibility for gene therapy in the coming years [43].

## 7. Ethics and Privacy

Ethical issues related to data privacy in AI research on inherited retinal dystrophies (IRD) are particularly important because ophthalmological data—including fundus images, OCT scans, and genotypes—can enable patient identification even if they are partially anonymized. This is due to the uniqueness of anatomical patterns and the high sensitivity of genetic data. The main risks include potential violations of the General Data Protection Regulation (GDPR), the risk of reconstructing personal data, the commercialization of data without the patient’s consent, and discrimination (e.g., insurance-based) based on a genetic or biomedical profile [34]

At the same time, the limited number of patients with IRD means that the available datasets are small and difficult to share between institutions, which makes it difficult to train reliable AI models. The lack of data diversity results in the risk of biased algorithms, and thus worse diagnostic effectiveness and inequality in access to precise diagnostics [44].

The recommendations include strengthening patient data protection through privacy techniques, such as federated learning and clear consent rules, as well as the development and use of synthetic data generated by GANs to increase the diversity of training sets and improve model quality. Clinical validation of such data and the creation of ethical standards, regulations, and explainability tools are necessary to ensure the transparent, safe, and reliable use of AI in the diagnosis of IRD. It is crucial to find a balance between protecting patients’ privacy and making high-quality data available for training AI models [20,44].

## 8. Conclusions

The application of deep learning in retinal image analysis may become a valuable tool to support the diagnosis of hereditary retinal diseases. AI enables genotype prediction, phenotype classification, and early-stage disease detection. The accuracy of genetic prediction in reviewed studies was high enough to support the diagnostic process before genetic testing, which can speed up and optimize patient care.

The use of artificial intelligence in IRD does not replace, but complements the classical genetic and ophthalmological examinations. Although the accuracy of AI predictions is high, there is still a need to verify the results using molecular methods. AI works best as a clinical decision support tool (CDSS), not as a standalone diagnostic system. The authors of most studies emphasize that AI shortens the time of diagnostics, but does not eliminate the need to confirm the results.

Several studies have shown that the effectiveness of models can decrease if they are trained on a population with limited genetic diversity or collected in one location. Therefore, building a global, diverse image and genetic databases is crucial for the further development of AI in IRD.

## Figures and Tables

**Table 1:** Application of AI in the diagnosis and monitoring gene therapy of retinal dystrophy.

AI Tool/Method	Clinical Application	Type of Input	Key Advantages
Convolutional networks (CNN)	Automatic analysis of OCT, FAF, OCTA images	Retinal images from OCT, FAF, OCTA	High precision detection of structural changes
Machine learning (ML)	Classification of IRD phenotypes, prediction of disease progression	Clinical, functional, genetic data	Integration of multidimensional data, possibility of prognosis
Deep learning (DL)	Automatic detection and segmentation of retinal lesions	OCT images, autofluorescence	Accurate segmentation and monitoring of lesions over time
Predictive algorithms	Predicting response to gene therapy	Clinical, genetic data	Personalizing therapy, optimizing treatment plan
Generative networks (GAN)	Synthesize images for training purposes	Retinal images	Increase training sets, improve quality of models

AI: artificial intelligence; OCT: Optical Coherence Tomography; FAF: Fundus autofluorescence; OCTA: Ocular Coherence Tomography Angiography; IRD: Inherited retinal dystrophies.

**Table 2:** Results from the included studies—summary.

Publication	AI Method	Sample Size	Network	Image Type	Classes	Accuracy (%)
Fujinami-Yokokawa et al., 2019 [14]	DL	n = 178	InceptionV3	OCT	*ABCA4*, *RP1L1*, *EYS*, Normal	90.9
Fujinami-Yokokawa et al., 2021 [15]	DL—CNN	n = 417	InceptionV3	fundus images, FAF	*ABCA4*, *EYS*, *RP1L1*, Normal	88.2 (fundus), 81.3 (FAF)
Kominami et al., 2025 [16]	CNN	n = 165	VGG16, Xception, DenseNet201, MobileNet	FAF, color fundus images	Retinitis Pigmentosa (the severity of RP)	63.75 (fundus), 87.50 (FAF)
Chen et al., 2021 [17]	DL	n = 8600	Inception V3, Inception Resnet V2, Xception	fundus images	Retinitis Pigmentosa	96.0
Miere et al., 2020 [18]	DL—CNN	n = 251	ResNet 101	FAF	Stargardt Disease, Retinitis Pigmentosa, Best Disease, Normal	95.0
Pontikos et al., 2025 [19]	DL	n = 133	Eye2Gene FAF Only/Eye2Gene	FAF, IR, OCT	63 distinct IRD genes	83.9 *

* top-five accuracy. DL: Deep Learning; CNN: Convolutional Neural Networks; OCT: Optical Coherence Tomography; FAF: Fundus Autofluorescence; IR: Infrared Reflectance Imaging; AI: Artificial Intelligence; *ABCA4*: ATP Binding Cassette Subfamily A Member 4; *RP1L1*: Retinitis pigmentosa 1-like 1 protein; *EYS*: EGF-Like Photoreceptor Maintenance Factor; VGG16: Very Deep Convolutional Networks; RP: Retinitis pigmentosa; IRD: Inherited retinal dystrophies.

**Table 3:** The effectiveness of AI models in diagnosing IRD based on OCT, FAF and fundus images.

Publication	Image Count	Classes	Image Type	Sens (%)	Spec (%)	Acc (%)
Fujinami-Yokokawa et al., 2019 [14]	19	*ABCA4*	OCT	100.00	100.00	100.00
Fujinami-Yokokawa et al., 2021 [15]	37		FAF	97.5	94.8	81.3
Shah et al., 2020 [20]	647	Stargardt	OCT	99.8	98.0	99.6
Miere et al., 2020 [18]	125		FAF	96.0	100.00	95.0
Chen et al., 2021 [17]	193	Retinitis Pigmentosa	fundus images	95.71	98.53	96.0
Miere et al., 2020 [18]	160		FAF	100.0	97.0	95.0

Sens: Sensitivity; Spec: Specificity; Acc: Accuracy; *ABCA4*: ATP-Binding Cassette Subfamily A Member 4; OCT: Optical Coherence Tomography; FAF: Fundus Autofluorescence.

**Table 4:** Review of studies using GAN in the context of diagnosing and monitoring IRD therapy.

GAN Model	Sample Size	Purpose of the Study	Application in IRD	Main Conclusions	AUC (95% CI)
StyleGAN2-ADA	n = 15,692	Generate synthetic FAF images in IRD patients	Fill in missing image data in IRD; train AI models for classification and lesion detection	Synthetic data improve accuracy of classification models, reduce risk of overfitting in small IRD datasets	-
RV-GAN (multi-scale GAN)	n = 15,120	Precise vascular segmentation in fundus images	Assessment of vascular lesions in IRD patients from fundus images	GAN provides high accuracy in segmentation, enabling monitoring of microangiopathy in IRD	0.988–0.991 *
AnoGAN	n = 8192	Detection of abnormalities in OCT images	Identification of subclinical retinal changes in IRD	Allows detection of irregular patterns that may indicate early or atypical forms of IRD	~0.85–0.90 *
f-AnoGAN	n = 70,000	Faster and more accurate detection of anomalies	Reduced OCT analysis time in patients with IRD	Improved speed and quality of anomaly detection compared to classic AnoGAN	~0.88–0.92 *
cGAN (conditional GAN)	n = 850	Translation of fundus image to fluorescein angiography (FA)	Generation of FA without the need for invasive examination, helpful in IRD with a vascular component	GAN can predict FA from plain fundus, which aids non-invasive IRD monitoring	0.91–0.95 *

* depending on the model used. GAN: Generative Adversarial Network; IRD: Inherited Retinal Disease; AUC: Area Under the Curve; CI: Confidence Interval; ADA: Adaptive Discriminator Augmentation; RV: Retinal Vasculature; FAF: Fundus Autofluorescence; AI: Artificial Intelligence; FA: Fluorescein Angiography.

## Data Availability

No new data were created or analyzed in this study. Data sharing is not applicable to this article.

## Supplementary Materials

The following supporting information can be downloaded at https://www.mdpi.com/article/10.3390/jpm15120605/s1. Table S1: PRISMA checklist.
